# Determination of Thymol in Commercial Formulation, Essential Oils, Traditional, and Ultrasound-Based Extracts of *Thymus vulgaris* and *Origanum vulgare* Using a Greener HPTLC Approach

**DOI:** 10.3390/molecules27041164

**Published:** 2022-02-09

**Authors:** Ahmed I. Foudah, Faiyaz Shakeel, Mohammed H. Alqarni, Abuzer Ali, Sultan Alshehri, Mohammed M. Ghoneim, Prawez Alam

**Affiliations:** 1 Department of Pharmacognosy, College of Pharmacy, Prince Sattam Bin Abdulaziz University, P.O. Box 173, Al-Kharj 11942, Saudi Arabia; a.foudah@psau.edu.sa (A.I.F.); m.alqarni@psau.edu.sa (M.H.A.); 2 Department of Pharmaceutics, College of Pharmacy, King Saud University, P.O. Box 2457, Riyadh 11451, Saudi Arabia; faiyazs@fastmail.fm (F.S.); salshehri1@ksu.edu.sa (S.A.); 3 Department of Pharmacognosy, College of Pharmacy, Taif University, P.O. Box 11099, Taif 21944, Saudi Arabia; abuali@tu.edu.sa; 4 Department of Pharmacy Practice, College of Pharmacy, AlMaarefa University, P.O. Box 71666, Ad Diriyah 13713, Saudi Arabia; mghoneim@mcst.edu.sa

**Keywords:** AGREE scale, essential oils, greener HPTLC, thymol, ultarsonication, validation

## Abstract

In the literature, greener analytical approaches for determining thymol in its commercial formulations, plant-based phytopharmaceuticals, and biological fluids are scarce. As a result, the goal of this study is to develop and validate a normal-phase “high-performance thin-layer chromatography (HPTLC)” method for determining thymol in commercial formulations, essential oils, traditional extracts (TE), and ultrasound-based extracts (UBE) of *Thymus vulgaris* and *Origanum vulgare* obtained from various geographical regions. The greener mobile phase for thymol analysis was a binary combination of cyclohexane and ethyl acetate (85:15, *v/v*). The derivatized densitometric analysis of thymol was carried out under visible mode at 530 nm utilizing anisaldehyde-sulfuric acid as a derivatizing/visualizing agent. In the 10–2000 ng/band range, the greener normal-phase HPTLC method was linear. Furthermore, for thymol analysis, the proposed analytical approach was simple, quick, inexpensive, accurate, precise, robust, sensitive, and greener. The thymol contents in commercial formulation were computed as 7.61% *w/w*. In general, the thymol contents were maximum in essential oils of *T. vulgaris* and *O. vulgare* compared to the other sample matrices studied. The thymol contents of TE of *T. vulgaris* and *O. vulgare* of different geographical regions were significantly low compared to their UBE extract. Using 12 distinct components of green analytical chemistry, the overall “analytical GREEnness (AGREE)” scale for the proposed analytical approach was computed 0.79, showing the good greener nature of the proposed analytical approach. Overall, the greener normal-phase HPTLC technique was found to be reliable for determining thymol in commercial formulations and plant-based phytopharmaceuticals.

## 1. Introduction

Thymol is a dietary monoterpene phenolic compound, which is isolated from a variety of plants, including *Thymus vulgaris* (family: Laminaceae), *T. serphyllum* (family: Laminaceae), *Monarda fustulosa* (family: Laminaceae), *Origanum vulgare* (family: Laminaceae), *Carum copticum* (family: Apiaceae), *Trachyspermum ammi* (family: Apiaceae), *Lippia multiflora* (family: Verbenaceae), *Centipeda minima* (family: Asteraceae), and *Nigella sativa* (family: Ranunculaceae) [1,2,3,4,5]. In literature, thymol had shown the variety of therapeutic activities such as analgesic, antioxidant, anti-inflammatory, antimicrobial, larvicidal, acaricidal, antimeishmanial, antiepileoptogenic, radioprotective, anti-hemolytic, and wound healing properties [6,7,8]. It is also used in a number of food and pharmaceutical items as a flavoring agent [8]. Due to wide pharmacological and pharmaceutical application of thymol, suitable and greener analytical approaches are required for its qualitative and quantitative analysis. 

For the examination of thymol in commercial formulations, essential oils, plant extracts, and biological fluids, many analytical techniques have been used, either alone or in combination with other phenolic phytochemicals. For the identification of thymol in its dosage forms and plant extracts, various visible-detection-based methods utilizing different derivatizing agents have been published [9,10,11,12]. The amount of thymol in thyme essential oil was also determined using a colorimetry method [13]. Some flow-injection spectrometry approaches are also reported for the analysis of thymol, which utilizes different derivatizing agents [14,15]. The determination of thymol has also been done using voltammetry methods [16,17]. A wide range of “high-performance liquid chromatography (HPLC)” approaches are utilized for determining thymol either alone or in combination with other phenolic compounds in dosage forms, skin permeation study samples, essential oils of different plants, and various plant extracts [18,19,20,21,22,23,24,25]. Various “high-performance thin-layer chromatography (HPTLC)” approaches have also been utilized for determining thymol either alone or in combination with other phenolic pharmaceuticals in Unani polyherbal formulations, essential oils, and plant extracts [26,27,28,29]. For the determination of thymol in essential oils, dosage forms, and plant extracts, various “gas-chromatography mass-spectrometry (GC-MS)” technique was used [30,31,32,33]. The determination of thymol in conjunction with eucalyptol, camphor, and menthol in Greek thyme honey was done using a GC-flame ionization detection (GC-FID) method [34]. Some GC-MS methods for determining thymol in various biological fluids, such as dairy cow plasma and milk, and human plasma, have also been published [35,36]. Some other techniques such as cerium oxide nanoparticles-based electrochemical sensor [37], liquid chromatography with fluorometric detection [38], and ultra-performance convergence chromatography [39] techniques have also been reported for the determination of thymol. 

After reviewing thymol analysis methods in the literature, we noticed that the safety and greener aspects of described analytical procedures had not been examined. In addition, the greener or environmental-friendly HPTLC approaches are not yet documented for thymol analysis in commercial formulations, essential oils, and plant-based phytopharmaceuticals. Greener HPTLC approaches offer many merits, which include “simplicity, economicity, low operation cost, short analysis time, parallel analysis of multiple samples, detection clarity, and reduction in environmental toxicity” compared to other analytical approaches [40,41,42,43]. Hence, a normal-phase HPTLC approach for thymol analysis was utilized in this study. Various environmentally friendly strategies are used to analyze the analytical procedures’ greener character [42,43,44,45,46,47]. However, “analytical GREEnness (AGREE)” approach applies all twelve principles/components of “green analytical chemistry (GAC)” for this purpose [46]. Therefore, the “AGREE metric approach” was utilized for the evaluation of greener nature of the greener normal-phase HPTLC approach [46]. Both cyclohexane (CY) and ethyl acetate (EtOAc) are categorized as green solvents according to GAC principle [40,48]. Accordingly, CY and EtOAc were used as green solvents for thymol analysis. This study involves the development and validation of a rapid, sensitive, and greener normal-phase HPTLC approach for the determination of thymol in commercial formulations, essential oils, traditional extracts (TE), and ultrasound-based extracts (UBE) of *T. vulgaris* and *O. vulagre* obtained from Saudi Arabia (SA), India (IND), and the United Kingdom (UK) based on all of these hypotheses. The greener characteristics of the present analytical approach was assessed utilizing “AGREE: The Analytical Greenness Calculator”. The proposed analytical approach for thymol analysis in commercial formulation, essential oils, TE, and UBE of *T. vulgaris* and *O. vulagre* was validated according to the “International Council for Harmonization (ICH) Q2 (R1)” recommendations [49].

## 2. Materials and Methods

### 2.1. Chemicals and Reagents

The standard thymol (purity > 98%) was obtained from “Sigma Aldrich (St. Louis, MO, USA)”. The chromatography-grades solvents such as methanol, CY, and EtOAc were obtained from “E-Merck (Darmstadt, Germany)”. Other reagents used in extraction and method development process were of analytical grades with high purity. The commercial herbal formulation containing the extracts of *T. vulgaris* and *O. vulgare* was purchased from a local pharmacy shop in “Al-Kharj, Saudi Arabia”. 

### 2.2. Plant Materials

The fresh leaves of *T. vulgaris* and *O. vulgare* were obtained from different geographical regions, including SA, IND, and UK. The identification for *T. vulgaris* and *O. vulgare* was done utilizing the identification key provided by the flora of Saudi Arabia. The voucher specimen for *T. vulgaris* and *O. vulgare* was deposited in “Herbarium of Department of Pharmacognosy, College of Pharmacy, Prince Sattam Bin Abdulaziz University, Al-Kharj, Saudi Arabia”. 

### 2.3. Chromatography and Analysis 

The “CAMAG HPTLC instrument (CAMAG, Muttenz, Switzerland)” was used to estimate thymol in its pure/bulk form, commercial formulation, essential oils of *T. vulgaris* and *O. vulgare*, TE and UBE of *T. vulgaris* and *O. vulgare* obtained from various geographical regions using normal-phase HPTLC. The analysis of thymol in normal-phase mode was carried out on “10 × 20 cm^2^ aluminum plates pre-coated with normal-phase silica gel 60 F254S plates (E-Merck, Darmstadt, Germany)”. Using a “CAMAG Automatic Sampler 4 (ATS4) applicator (CAMAG, Geneva, Switzerland)”, the samples to the normal-phase TLC plates were spotted as 6 mm bands. The “CAMAG microliter Syringe (Hamilton, Bonaduz, Switzerland)” was used as a sampling applicator. In normal-phase mode, the application rate for thymol analysis was set at 150 nL/s. The normal-phase TLC plates were developed at a distance of 80 mm in a “Automatic Developing Chamber 2 (ADC 2) (CAMAG, Muttenz, Switzerland)”. The greener mobile phase for thymol analysis was CY-EtOAc (85:15, *v/v*). For 30 min at 22 °C, the development chamber was saturated using CY-EtOAc (85:15, *v/v*) vapors. The slit dimensions were set at 4 × 0.45 mm^2^ and scanning rate was made constant at 20 mm/s. 

### 2.4. Derivatization and Densitometry Scanning 

After development of normal-phase TLC plates in ADC 2, the procedure was derivatized by spraying with anisaldehyde-sulfuric acid visualizing agent using the CAMAG glass reagent spray technique. Then, the normal-phase TLC plates were heated for about 10 min at 110 °C. After 30 min, thymol was analyzed using a CAMAG TLC scanner III attached to the “WinCAT’s (v. 1.2.3., CAMAG, Muttenz, Switzerland)” software and the plates were scanned at 530 nm. At least three replicates (*n* = 3) of each analysis were carried out.

### 2.5. Preparation of Thymol Standard Solutions for Calibration and Quality Control (QC) 

The appropriate amount of thymol (10 mg) was mixed in 100 mL of CY-EtOAc (85:15, *v/v*) greener mobile phase to form a stock solution of thymol with a concentration of 100 µg/mL. Various volumes of this stock solution were diluted further with CY-EtOAc (85:15, *v/v*) greener mobile phase to obtain thymol concentrations in the 10–2000 ng/band range. Thymol solutions of various concentrations were produced and applied to normal-phase TLC plates. The derivatized densitometric response for thymol was evaluated for each thymol solution using the greener normal-phase HPTLC approach. Thymol concentrations vs. measured derivatized densitometric response was plotted to obtain thymol calibration curve. In addition, three QC samples, including low QC (LQC; 50 ng/band), middle QC (MQC; 500 ng/band), and high QC (HQC; 2000 ng/band) were prepared separately for the evaluation of several validation parameters for the greener normal-phase HPTLC test.

### 2.6. Sample Processing of Thymol from Commercial Formulation 

The commercial formulation of thymol contains different plant extracts including the extracts of *T. vulgaris* and *O. vulgare*. Maceration in methanol (3 × 100 mL) at room temperature was used to extract samples (2.5 g) from commercial herbal preparations. Whatman filter paper (No. 41) was used to filter each sample. A rotary vacuum evaporator at 40 °C was used to evaporate the solvent from the extract of a commercial herbal product under reduced pressure. The concentrated extracts from commercial herbal formulations were then reconstituted with 25 mL of methanol and kept in the refrigerator until further testing. Three times (*n* = 3) this technique was repeated. This solution was used as a test solution to determine the presence of thymol in a commercial herbal preparation. 

### 2.7. Isolation of the Essential Oil from T. vulgaris and O. vulgare of Different Geographical Regions 

The essential oil of *T. vulgaris* and *O. vulgare* from the IND, SA, and UK regions was isolated using the Egyptian Pharmacopoeia’s conventional hydro-distillation process. Total of 250 g of fresh leaves of *T. vulgaris* and *O. vulgare* from IND, SA, and UK areas were used to extract essential oils using the Clevenger trap equipment. For around 8 h distillations, the needed amount of each plant was blended with 1000 mL of water. Dichloromethane was used to capture the oil layer and water separation (3 × 50 mL). The organic layer was further condensed using a rotating vacuum evaporator to isolate the pure essential oil from each plant. This technique was repeated three times (*n* = 3). This solution was used as a test solution to determine the presence of thymol in essential oils of *T. vulgaris* and *O. vulagre* of IND, SA, and UK regions using the greener HPTLC approach.

### 2.8. Traditional Extraction of Thymol from T. vulgaris and O. vulgare of Different Geographical Regions 

Fresh green leaves of *T. vulgaris* and *O. vulagre* were collected from different geographical regions, including IND, SA, and UK and air dried. Maceration with methanol (3 × 100 mL) at room temperature was used to extract 5 g of powdered dried leaves of *T. vulgaris* and *O. vulagre* from the IND, SA, and UK regions. Whatman filter paper (No. 41) was used to filter each sample. The solvent from *T. vulgaris* and *O. vulagre* extracts from different geographical regions was evaporated separately at 40 °C using a rotary vacuum evaporator under reduced pressure. The concentrated extracts were then reconstituted with 50 mL of methanol from various geographical regions. This conventional extraction (TE) test (*n* = 3) was done in triplicate. The prepared solution was utilized as a test solution for the determination of thymol in methanolic TE of *T. vulgaris* and *O. vulagre* of IND, SA, and UK regions using the greener normal-phase HPTLC approach. 

### 2.9. Ultrasound-Based Extraction of Thymol from T. vulgaris and O. vulgare of Different Geographical Regions 

The dried leaves of *T. vulgaris* and *O. vulagre* from the IND, SA, and UK regions were ultrasound-based extracted (UBE) using “Bransonic series ultrasound vibrations (Model CPX5800H-E; NJ, USA)”. About 50 g of powdered dried leaves of *T. vulgaris* and *O. vulagre* from the IND, SA, and UK regions were accurately weighed and extracted with 100 mL of methanol. The solvent was removed using a rotary vacuum evaporator, and the residue was dissolved in 50 mL of methanol. For roughly an hour, the reconstituted solution was ultrasonicated at 50 °C. Triplicates (*n* = 3) were also used for the UBE. Using the greener normal-phase HPTLC technique, the resulting solution was used as a test solution for determining thymol in UBE of *T. vulgaris* and *O. vulagre* from the IND, SA, and UK regions. 

### 2.10. Validation Studies 

The greener normal-phase HPTLC technique for thymol analysis was verified for a variety of parameters in accordance with ICH-Q2-R1 requirements [49]. Plotting thymol concentrations vs. observed densitometric response was used to determine thymol linearity. Thymol linearity was investigated at ten different QC solutions of 10, 20, 50, 100, 200, 300, 400, 500, 1000, and 2000 ng/band for the greener HPTLC approach. The verification of linear model assumption was performed in terms of significance of estimators, normality of the residues, the autocorrelation of the random component, and heteroscedasticity using Regressit software. “Retardation factor (R_f_), asymmetry factor (As), and number of theoretical plates per meter (N/m)” were utilized to test system suitability for the greener HPTLC approach. The “R_f_, As, and N/m” values were assessed at MCQ (500 ng/band) by adopting their reported formulae [50].

The percent recovery was utilized to estimate the accuracy of the greener normal phase HPTLC assay. At three different levels, such as LQC (50 ng/band), MQC (500 ng/band), and HQC (2000 ng/band), the percent recovery of thymol was determined.

The intra/intermediate precision rating was awarded to the proposed analytical approach. By measuring thymol at LQC, MQC, and HQC on the same day, the proposed analytical approach was utilized to assess intraday variance. On three distinct days, the proposed analytical approach was performed to detect intermediate variation by measuring thymol at LQC, MQC, and HQC [49].

A slight planned alteration in the greener mobile phase for the proposed analytical approach was utilized to test the robustness. The initial CY-EtOAc (85:15, *v/v*) greener mobile phase was converted to CY-EtOAc (87:13, *v/v*) and CY-EtOAc (83:17, *v/v*) green mobile phases for the robustness assessment, with the needed changes in densitometric response and R_f_ values specified [49].

The sensitivity for the proposed analytical approach was assessed as “detection (LOD) and quantification (LOQ) limits” utilizing a reported standard deviation technique. The “LOD and LOQ” of thymol for the greener normal-phase HPTLC approach was determined by its standard formulae [49,50].

By comparing the R_f_ values and UV absorption spectra of thymol in commercial formulation, essential oils of *T. vulgaris* and *O. vulgare*, TE and UBE of *T. vulgaris* and *O. vulgare* with those of standard thymol for the greener normal-phase HPTLC approach, the peak purity/specificity was evaluated.

### 2.11. Analysis of Thymol in Commercial Formulation, Essential Oils, TE, and UBE of Different Geographical Regions 

The densitometric responses of the prepared solutions of commercial formulation, essential oils, TE, and UBE of *T. vulgaris* and *O. vulgare* of different geographical regions were recorded on normal-phase TLC plates. The thymol contents in each sample matrices were calculated utilizing thymol calibration curve for the greener normal-phase HPTLC approach.

### 2.12. Greenness Assessment 

The “AGREE metric methodology” [46] was utilized to investigate the greener nature of the greener normal-phase HPTLC approach. “AGREE: The Analytical Greenness Calculator (version 0.5, Gdansk University of Technology, Gdansk, Poland, 2020)” was used to calculate the AGREE scales (0.0–1.0) for the proposed analytical approach.

## 3. Results and Discussion 

### 3.1. Method Development

In method development process, the first step is to optimize chromatographic conditions for the analysis of drug. During method development step, test samples cannot be applied because chromatographic conditions are not optimized. After optimizing chromatographic parameters, test samples can be applied. The nature and type of plates for standard and test samples were same for standard and test samples. However, the samples were applied on different time. Despite several reported pharmaceutical assays of thymol analysis, the greener normal-phase HPTLC assays for thymol analysis are scarce in literature. As a result, the development of a greener normal-phase HPTLC approach for thymol analysis in commercial formulations, essential oils of *T. vulgaris* and *O. vulgare* from various geographical regions, TE of *T. vulgaris* and *O. vulgare* from various geographical regions, and UBE of *T. vulgaris* and *O. vulgare* from various geographical regions are all part of this research.

**Figure 1:**
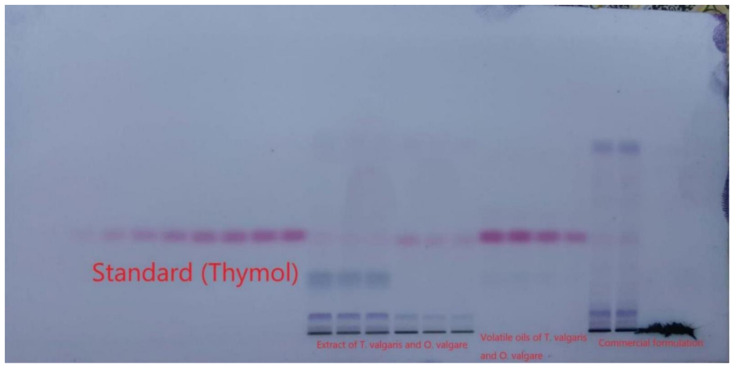
Thin-layer chromatography (TLC)-plate for standard thymol, essential oils, extracts, and commercial formulation developed using CY-EtOAc (85:15, ***v/v***) as the greener mobile phase after derivatization using anisaldehyde-sulfuric acid under chamber saturation conditions for the greener normal-phase HPTLC assay.

**Figure 2:**
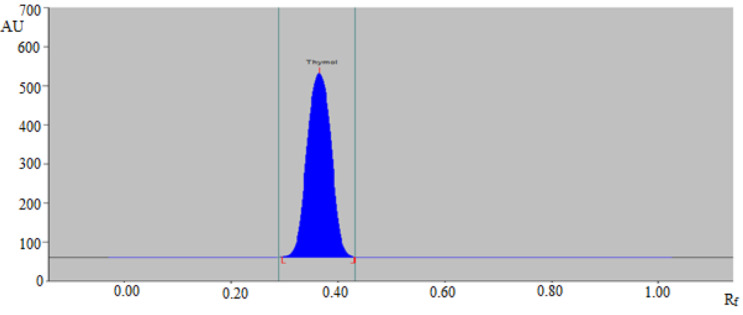
Normal-phase HPTLC densitogram of 500 ng/band concentration of standard thymol for the greener normal-phase HPTLC approach.

For the densitometric estimation of thymol, different amounts of CY and EtOAc, such as CY-EtOAc (50:50, ***v/v***), CY-EtOAc (55:45, ***v/v***), CY-EtOAc (60:40, ***v/v***), CY-EtOAc (65:35, ***v/v***), CY-EtOAc (70:30, ***v/v***), CY-EtOAc (75:25, ***v/v***), CY-EtOAc (80:20, ***v/v***), CY-EtOAc (85:15, ***v/v***), and CY-EtOAc (90:10, ***v/v***) were evaluated as the greener mobile phases for the development of a reliable band for thymol estimation. All greener mobile phases were established using the chamber saturation conditions as summarized in Figure 1. From the data recorded, it was noticed that CY-EtOAc (50:50, ***v/v***), CY-EtOAc (55:45, ***v/v***), CY-EtOAc (60:40, ***v/v***), CY-EtOAc (65:35, ***v/v***), CY-EtOAc (70:30, ***v/v***), CY-EtOAc (75:25, ***v/v***), CY-EtOAc (80:20, ***v/v***), and CY-EtOAc (90:10, ***v/v***) greener mobile phases offered a poor chromatogram of thymol with an unacceptable As value (As = 1.21). However, the CY-EtOAc (85:15, ***v/v***) greener mobile phase offered a well-separated peak of thymol at R_f_ = 0.37 ± 0.01 with a reliable As value (As = 1.05 ± 0.03) (Figure 2). Hence, the CY-EtOAc (85:15, ***v/v***) was selected as the greener mobile phase for thymol estimation in its commercial formulation, essential oils, TE, and UBE of different geographical regions. The highest densitometric response was recorded at 530 nm following derivatization with anisaldehyde-sulfuric acid for the proposed analytical approach, and the UV-spectral bands for the proposed analytical approach were calculated in densitometric mode. As a result, the complete thymol quantification was done at 530 nm. 

### 3.2. Validation Studies

The proposed analytical approach for thymol analysis was validated for several parameters [48]. Table 1 summarizes the results of the linearity study of the thymol calibration plot for the proposed analytical approach. For the proposed analytical approach, the thymol calibration plot was linear in the 10–2000 ng/band range. For the proposed analytical approach, the determination coefficient (R^2^) and regression coefficient (R) for thymol were estimated to be 0.9991 and 0.9995, respectively. Both lower and higher concentrations were considered for the evaluation of linearity range. The entire range of concentrations was selected where R^2^ value was maintained greater than 0.99. Up to 10–2000 ng/band range, the R^2^ value was greater than 0.99. Hence, this linearity range was selected. The linear model for thymol calibration curve was significant (*p* < 0.05). The residues were normally distributed. Durbin-Watson statistic value for autocorrelation was predicted as 0.739, indicating positive autocorrelation. The *p* value for heteroscedasticity test was estimated to be greater than 0.05, indicating no significance evidence of heteroscedasticity. These results revealed a solid linear relationship between thymol content and densitometric response.

The parameters for the proposed analytical method’s system suitability were evaluated at MQC (500 ng/band). For the greener normal-phase HPTLC approach, the “R_f_, As, and N/m” values were found to be 0.37 ± 0.01, 1.05 ± 0.03, and 4694 ± 2.87, respectively. These data showed that the greener normal-phase HPTLC approach was suitable for thymol analysis in commercial formulation, essential oils, TE, and UBE of different geographical regions. 

Table 2 summarizes the findings of the accuracy evaluation for the proposed analytical approach. At three different QC levels, the percent thymol recovery for the greener normal-phase HPTLC approach was estimated to be between 98.42 and 101.11 percent. These values of percent thymol recoveries showed the accuracy of the greener normal-phase HPTLC approach for thymol analysis in its commercial formulation, essential oils, TE, and UBE of different geographical regions.

The precision for the greener normal-phase HPTLC approach was assessed as the percent of the coefficient of variation (% CV) and results are summarized in Table 3. The % CVs of thymol for the greener normal-phase HPTLC approach were recorded as 0.93%, 0.64%, and 0.34% at LQC, MQC, and HQC, respectively for the intra-assay precision. The % CVs of thymol for the greener normal-phase HPTLC approach were obtained as 0.98%, 0.62%, and 0.36% at LQC, MQC, and HQC, respectively for the inter-assay precision. These data suggested the precision of the greener normal-phase HPTLC approach for thymol analysis in its commercial formulation, essential oils, TE, and UBE of different geographical regions.

Table 4 summarizes the findings of the robustness assessment for the proposed analytical approach. For the greener normal-phase HPTLC approach, the percent CVs for the robustness assessment were assessed to be 0.66–0.76 percent. The R_f_ values were recorded in the range of 0.36–0.38. The small variations in the R_f_ values of thymol and lower % CVs indicated the robustness of the greener normal-phase HPTLC approach for thymol analysis in its commercial formulation, essential oils, TE, and UBE of different geographical regions. 

The sensitivity for the proposed analytical approach was assessed as “LOD and LOQ” and their predicted values are included in Table 1. The “LOD and LOQ” for the proposed analytical approach were recorded as 10.31 *±* 0.24 and 30.93 *±* 0.72 ng/band, respectively for thymol analysis. The obtained value of LOQ was three times of LOD. In addition, the LOQ was predicted within the calibration range of the proposed analytical method. These data of “LOD and LOQ” for the proposed analytical approach showed the sensitivity for thymol analysis in its commercial formulation, essential oils, TE, and UBE of different geographical regions. 

**Figure 3:**
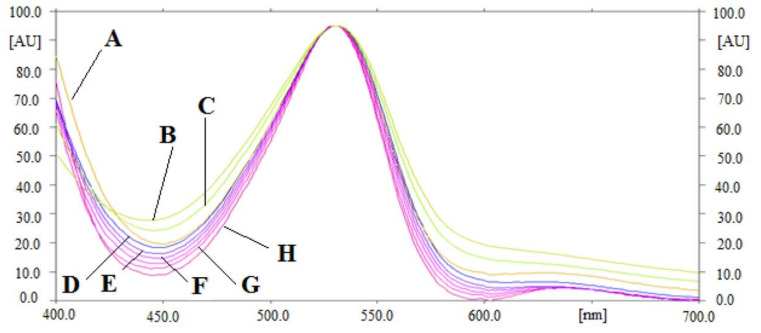
UV absorption spectra of (A) standard thymol, (B) essentail oil of *Origanum vulgare* (SA), (C) essentail oil of *Thymus vulgaris* (SA), (D) UBE of *Origanum vulgare* (SA), (E) UBE of *Thymus vulgaris* (SA), (F) TE of *Origanum vulgare* (SA), (G) TE of *Thymus vulgaris* (SA), and (H) commercial formulation, superimposed.

By comparing the superimposed UV-absorption spectra of thymol in commercial formulation, essential oils of *T. vulgaris* and *O. vulgare*, TE of *T. vulgaris* and *O. vulgare*, and UBE of *T. vulgaris* and *O. vulgare* with those of pure thymol, the peak purity/specificity for the proposed analytical approach was assessed. Figure 3 shows the UV-absorption spectra of pure thymol and thymol in commercial formulation, essential oils of *T. vulgaris* and *O. vulgare*, TE of *T. vulgaris* and *O. vulgare*, and UBE of *T. vulgaris* and *O. vulgare*, superimposed. The highest densitometric response for thymol in pure form and commercial formulation, essential oils of *T. vulgaris* and *O. vulgare*, TE of *T. vulgaris* and *O. vulgare*, and UBE of *T. vulgaris* and *O. vulgare* was found at 530 nm after derivatization with anisaldehyde-sulfuric acid at visible mode. The peak purity/specificity for the proposed analytical approach was suggested by the similar UV-absorption spectra, R_f_ values, and detection wavelength of thymol in pure thymol, commercial formulation, essential oils of *T. vulgaris* and *O. vulgare*, TE of *T. vulgaris* and *O. vulgare*, and UBE of *T. vulgaris* and *O. vulgare*.

### 3.3. Analysis of Thymol in Commercial Formulation, Essential oils, TE, and UBE of Different Geographical Regions 

**Figure 4:**
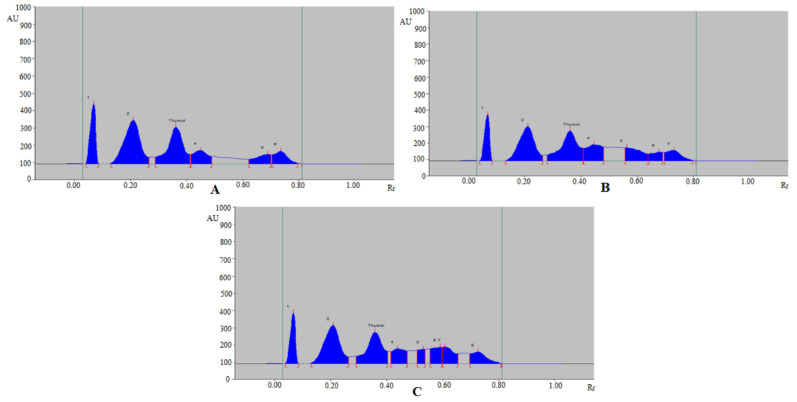
Representative HPTLC chromatograms of thymol in TE of (**A**) *T. vulgaris* (SA), (**B**) *T. vulgaris* (IND), and (**C**) *T. vulgaris* (UK).

**Figure 5:**
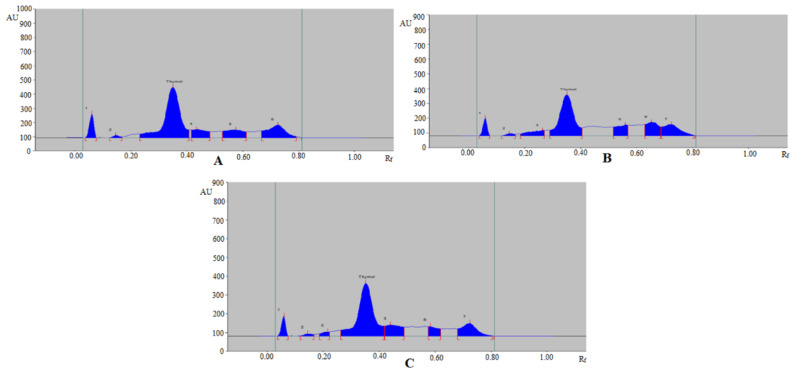
Representative HPTLC chromatograms of thymol in TE of (**A**) *O. vulgare* (SA), (**B**) *O. vulgare* (IND), and (**C**) *O. vulgare* (UK).

**Figure 6:**
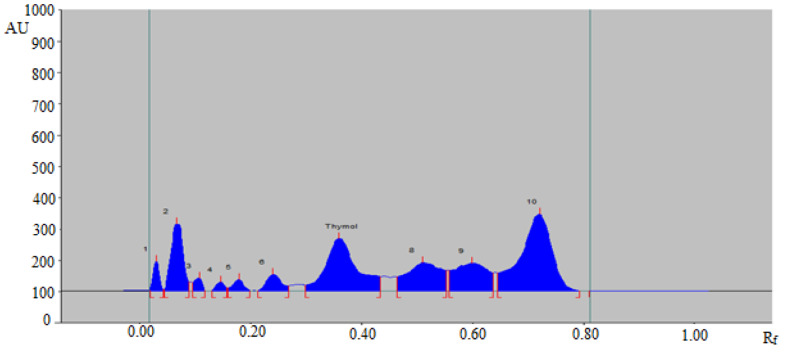
Representative HPTLC chromatograms of thymol in its commercial formulation.

Based on acceptable validation parameters, the proposed analytical method was applied in the determination of thymol in commercial formulation, TE, and UBE extracts of *T. vulgaris* and *O. vulgare* obtained from different geographical regions. The HPTLC peak of thymol from commercial formulation, essential oils, TE, and UBE of *T. vulgaris* and *O. vulgare* of different geographical regions was identified by obtaining its single TLC spot at R_f_ = 0.37 ± 0.01 for thymol with that of pure thymol. The representative HPTLC chromatograms of thymol in TE of *T. vulgaris* from SA, IND, and UK region are summarized in Figure 4, which showed identical peak of thymol with that of pure thymol. In addition, five, six, and seven additional peaks were also recorded in TE of *T. vulgaris* from SA (Figure 4A), IND (Figure 4B), and UK (Figure 4C) region, respectively. The HPTLC chromatograms of thymol in TE of *O. vulgare* from SA, IND, and UK region are summarized in Figure 5, which also showed identical peak of thymol with that of pure thymol. In addition, five, six, and six additional peaks were also recorded in TE of *O. vulgare* from SA (Figure 5A), IND (Figure 5B), and UK region (Figure 5C), respectively. The densitometry chromatogram of thymol in commercial formulation is summarized in Figure 6, which also presented identical peak of thymol with that of pure thymol. In addition, nine additional peaks were also observed in the commercial formulation of thymol. The presence of additional peaks in different sample matrices indicated that the greener normal-phase HPTLC approach can be effectively utilized in the determination of thymol in the presence of impurities/different compounds. The content of thymol in commercial formulation, essential oil, TE, and UBE of *T. vulgaris* and *O. vulgare* of different geographical regions was calculated using thymol calibration curve, and results are tabulated in Table 5. 

The content of thymol in its commercial formulation was determined as 7.61 ± 0.62% *w/w* using the proposed analytical approach. The amount of thymol in essential oils of *T. vulgaris* of SA, IND, UK origin was determined as 40.72 ± 1.84, 31.67 ± 1.64, and 33.81 ± 2.12% *w/w*, respectively using the proposed analytical approach. The amount of thymol in essential oils of *O. vulgare* of SA, IND, UK origin was determined as 54.32 ± 2.64, 41.10 ± 1.34, and 45.60 ± 1.34% *w/w*, respectively using the proposed analytical approach. The amount of thymol in TE of *T. vulgaris* of SA, IND, UK origin was determined as 8.60 ± 0.76, 5.44 ± 0.32, and 6.31 ± 0.41% *w/w*, respectively using the proposed analytical approach. The content of thymol in TE of *O. vulgare* of SA, IND, UK origin was determined as 18.43 ± 0.91, 12.96 ± 0.84, and 13.58 ± 0.87% *w/w*, respectively using the proposed analytical approach. The content of thymol in UBE of *T. vulgaris* of SA, IND, UK origin was determined as 10.55 ± 0.79, 6.85 ± 0.37, and 8.49 ± 0.41% *w/w*, respectively using the proposed analytical approach. The amount of thymol in UBE of *O. vulgare* of SA, IND, UK origin was determined as 22.08 ± 1.09, 15.38 ± 0.68, and 15.93 ± 0.72% *w/w*, respectively using the proposed analytical approach. In general, the amounts of thymol were higher in essential oils of each plant compared to the other sample matrices studied. Compared to TE, the amount of thymol was significantly higher in UBE of all sample matrices studied (*p* < 0.05). Based on these observations, the UBE procedure for the extraction of thymol has been considered as superior over its TE procedure.

### 3.4. Greenness Assessment

**Figure 7:**
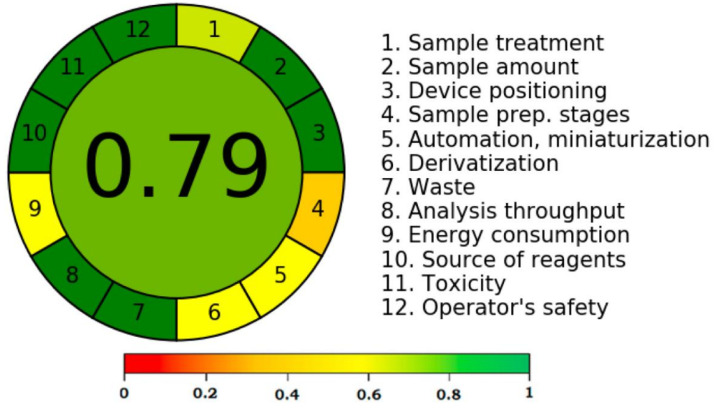
Analytical GREEnness (AGREE) scale for the greener normal-phase HPTLC approach.

**Figure 8:**
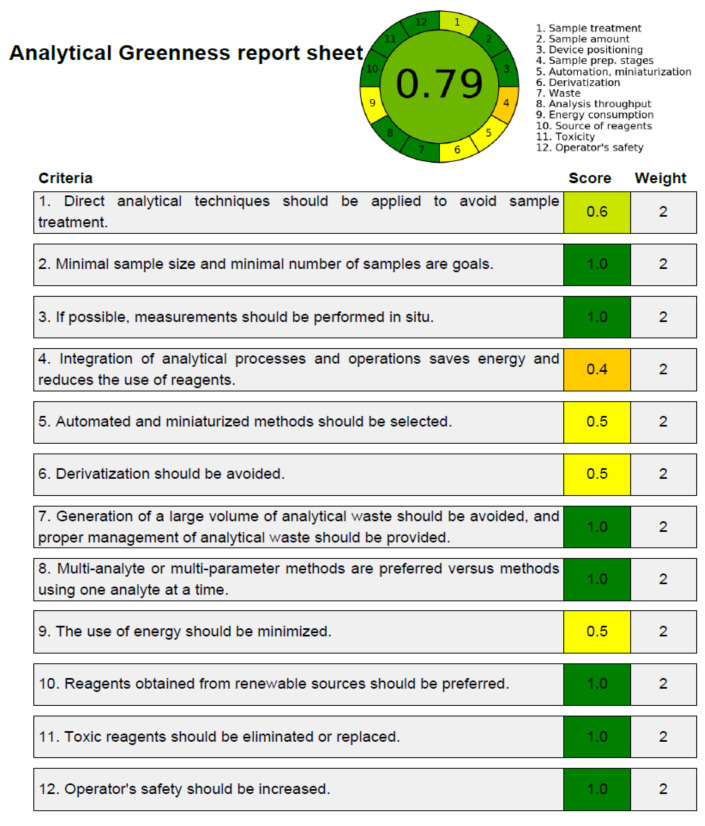
AGREE scale sheet for the greener normal-phase HPTLC approach of thymol, demonstrating the AGREE scale for 12 different components/principles of GAC.

Despite several reported approaches for determining greener nature of analytical procedures [42,43,44,45,46,47], only the “AGREE methodology” [46] employs all 12 GAC principles. Accordingly, the greenness nature of the present approach was assessed utilizing “AGREE Calculator”. Figure 7 depicts the overall AGREE scale for the present analytical approach. Figure 8 lists the AGREE report sheet and AGREE score for each particular GAC principle. The total AGREE scale for the proposed analytical approach was computed as 0.79, suggesting that the proposed analytical approach for thymol analysis is extremely green.

## 4. Conclusions

This study describes the invention and validation of a normal-phase HPTLC approach for determining thymol in commercial formulations, essential oils, TE, and UBE of *T. vulgaris* and *O. vulgaris* collected from SA, IND, and the UK. The proposed analytical approach was validated as per ICH recommendations. The proposed analytical approach was sensitive, rapid, and greener for thymol analysis. The contents of thymol were maximum in essential oils of both plants compared to the other sample matrices. In addition, the amount of thymol in *T. vulgaris* and *O. vulgare* UBE was much larger than in their TE process. Hence, UBE for thymol extraction is considered as superior over its TE procedures. The overall AGREE scale for the proposed analytical approach indicated its excellent greener profile for thymol analysis. These findings show that the proposed analytical approach can be used to analyze thymol in commercial items as well as a wide range of plant-based extracts and phytopharmaceuticals.

## Figures and Tables

**Table 1:** Results of the linear regression analysis for the analysis of thymol using the greener HPTLC assay ^a^.

Parameters	Values ^a^
Linearity range (ng/band)	10–2000
Regression equation	y = 14.214x + 663.79
R^2^	0.9991
R	0.9995
Slope ± SD	14.214 ± 0.84000
Intercept ± SD	663.79 ± 8.3900
Standard error of slope	0.34299
Standard error of intercept	3.4258
95% confidence interval of slope	12.738–15.689
95% confidence interval of intercept	649.04–678.53
LOD ± SD (ng/band)	10.31 ± 0.24
LOQ ± SD (ng/band)	30.93 ± 0.72

^a^ Mean ± SD; *n* = 6; LOD: limit of detection; LOQ: limit of quantification.

**Table 2:** The % thymol recoveries for the greener normal-phase HPTLC method ^a^.

Conc. (ng/band)	Conc. Found (ng/band) ± SD	Recovery (%)	CV (%)
50	49.21 ± 0.54	98.42	1.09
500	494.65 ± 3.14	98.93	0.63
2000	2022.32 ± 7.21	101.11	0.35

^a^ Mean ± SD; *n* = 6.

**Table 3:** Evaluation of precision of thymol for the greener normal-phase HPTLC method ^a^.

Conc. (ng/band)	Intraday Precision			Interday Precision		
	Conc. (ng/band) ± SD	Standard Error	CV (%)	Conc. (ng/band) ± SD	Standard Error	CV (%)
50	51.23 ± 0.48	0.19	0.93	50.68 ± 0.50	0.20	0.98
500	504.64 ± 3.25	1.32	0.64	493.65 ± 3.08	1.25	0.62
2000	1987.24 ± 6.84	2.79	0.34	2024.21 ± 7.38	3.01	0.36

^a^ Mean ± SD; *n* = 6.

**Table 4:** Results of robustness assessment of thymol for the proposed analytical approach ^a^.

Conc. (ng/band)	Mobile Phase Composition (CY-EtOAc)			Results		
	Original	Used	Level	Conc. (ng/band) ± SD	% CV	R_f_
		87:13	+2.0	485.31 ± 3.24	0.66	0.36
500	85:15	85:15	0.0	491.31 ± 3.54	0.72	0.37
		83:17	−2.0	511.25 ± 3.91	0.76	0.38

^a^ Mean ± SD; *n* = 6.

**Table 5:** Determination of thymol in commercial formulation, essential oil, and methanolic extracts extracted by traditional and ultrasound methods using a greener normal-phase HPTLC approach (mean *±* SD; *n* = 3).

Samples	Traditional Extraction	Ultrasound-Based Extraction
Amount of Thymol (%*w*/*w*)		
Commercial formulation	7.61 ± 0.62	NA *
Essential oil of *T. vulgaris* (SA)	40.72 ± 1.84	NA *
Essential oil of *T. vulgaris* (IND)	31.67 ± 1.64	NA *
Essential oil of *T. vulgaris* (UK)	33.81 ± 2.12	NA *
Essential oil of *O. vulgare* (SA)	54.32 ± 2.64	NA *
Essential oil of *O. vulgare* (IND)	41.10 ± 1.48	NA *
Essential oil of *O. vulgare* (UK)	45.60 ± 1.34	NA *
Extract of *T. vulgaris* (SA)	8.60 ± 0.76	10.55 ± 0.79
Extract of *T. vulgaris* (IND)	5.44 ± 0.32	6.85 ± 0.37
Extract of *T. vulgaris* (UK)	6.31 ± 0.41	8.49 ± 0.41
Extract of *O. vulgare* (SA)	18.43 ± 0.91	22.08 ± 1.09
Extract of *O. vulgare* (IND)	12.96 ± 0.84	15.38 ± 0.68
Extract of *O. vulgare* (UK)	13.58 ± 0.87	15.93 ± 0.72

* Not applicable.

## Data Availability

This study did not report any data.

## Sample Availability

Samples of the compounds thymol are available from the authors.
